# Advancing breast cancer relapse prediction with radiomics and neural networks: a clinically interpretable framework

**DOI:** 10.3389/fonc.2025.1593806

**Published:** 2025-09-15

**Authors:** Adnan Khalid, Muhammad Mursil, Carlos López Pablo, Ramon Bosch, Domenec Puig, Hatem A. Rashwan

**Affiliations:** ^1^ Department of Computer Engineering and Mathematics, Universitat Rovira i Virgili, Tarragona, Spain; ^2^ Hospital de Tortosa Verge de la Cinta, Institut Catala de la Salut, Tortosa, Spain

**Keywords:** breast cancer recurrence, diagnosis, personalized treatment, relapse, clinical features, radiomic features

## Abstract

Early assessment of breast cancer relapse can significantly impact survival rates and overall oncological outcomes, highlighting the need to use sophisticated diagnostic strategies in clinical trials. This work utilizes clinically relevant radiomic features extracted from digital mammograms to develop a deep learning-based model for forecasting breast cancer relapse. Features, including tumor size, shape, margin characteristics, molecular subtype, and breast density, were systematically extracted from our private, in-house dataset, providing a comprehensive representation of intrinsic tumor properties and assisting in relapse prediction. The predictive model demonstrated outstanding performance with an average area under the curve (AUC) of 0.957, highlighting its effectiveness in identifying possible relapse. This approach not only underscores the abilities of radiomics in enhancing the granularity of tumor assessment but also assists in identifying cancer recurrence during the treatment stage, promising significant strides toward personalized cancer therapy.

## 1 Introduction

Breast cancer (BC), as the most prevalent malignancy among women globally, presents significant challenges not only in terms of diagnosis but also in monitoring for recurrence. BC relapse has substantial prognostic implications; recurrence frequently indicates a worse prognosis and necessitates more aggressive treatment approaches Graham et al. (1). Targeted adjuvant therapies play a pivotal role in mitigating the risk of relapse based on key biomarkers PR (progesterone receptor), ER (estrogen receptor), and HER2 (human epidermal growth factor receptor Type 2) Boyages et al. (2). At least 85% of patients might receive unnecessary treatment if adjuvant chemotherapy were given to all patients without discrimination Hassett et al. (3). According to Dinan et al. (4), the only multi-gene test supported by the American Society of Clinical Oncology (ASCO) and the National Comprehensive Cancer Network (NCCN) for determining the risk of breast cancer recurrence is Oncotype DX, which was approved by the US FDA in 2005. Although it provides essential prognostic information Sparano et al. (5), its widespread use is limited because of the high cost of around $4,000 per patient. Thus, the development of a non-invasive and precise method for predicting BC recurrence risk holds significant clinical importance.

Advancements in digital mammography and radiomics have significantly enhanced our ability to detect early signs of recurrence through non-invasive methods Mao et al. (6). The integration of radiomics into BC diagnosis has revolutionized the potential of mammography, converting standard imaging into a rich data source that can enlighten hidden disease characteristics and predict clinical outcomes Lambin et al. (7). Quantitative data extraction from mammograms allows characterization of lesion size and shape, and may provide indirect indicators related to margin characteristics. All these characteristics have a relation with molecular subtypes, although these require further validation Ma et al. (8). Breast density has emerged as one of the most critical risk factors for cancer development and recurrence. Women with high breast density are at risk of recurrence following breast-conserving surgery and radiotherapy, as well as an increased likelihood of invasive BC after surgery for ductal carcinoma *in situ* Park et al (9) Habel et al. (10). BC molecular subtypes (Luminal A, Luminal B, Triple Negative (TN), and HER-2) have significantly influenced recurrence patterns. Notably, women over 50 with HER-2 positive and TN BC subtypes are at a higher risk of local recurrence compared to those with luminal cancers after surgery Ghose et al. (11). Early and accurate prediction of recurrence in these patients from clinical mammograms could facilitate more aggressive and tailored interventions.

Deep learning (DL) has substantially advanced the radiomics field Qi et al. (12), enhancing feature extraction and analysis from medical images for predicting breast cancer relapse Mao et al. (6)Dasgupta et al. (13). Recent work employing DL on mammograms has significantly improved breast density estimation Khara et al. (14) Biroš et al. (15). Techniques such as conditional Generative Adversarial Networks (cGANs) for segmenting high-density areas, when integrated with Fully Convolutional Networks (FCNs), have established benchmarks in density estimation Anyfantis et al. (16)Saffari et al. (17). They achieve more than 98% accuracy in classifying breast densities into precise categories (A: entirely fatty, B: scattered areas of fibro-glandular density, C: heterogeneously dense, D: extremely dense) is crucial for assessing cancer risk and predicting recurrence likelihood. Similarly, deep learning models such as Single Shot Detectors (SSD) Ruban et al. (18) and YOLO Kebede et al. (19) have proven effective in identifying and classifying tumor regions within mammograms Singh and Alam (20). These models efficiently delineate regions of interest (ROIs) that display characteristics indicative of malignancies. After identifying the regions of interest (ROIs) and utilizing advanced segmentation techniques Baccouche et al. (21) for precise tumor boundary delineation, which enables detailed analysis of the tumor region Li et al. (22). Feature extraction methods Qi et al. (12) are then applied to further classify the tumor by shape Singh et al. (23), margin, and molecular subtypes Rayamaihi et al. (24) Ma et al. (25). This level of detailed analysis aids in tailoring personalized treatment plans based on the radiomic signatures extracted from the tumor region.

**Figure 1:**
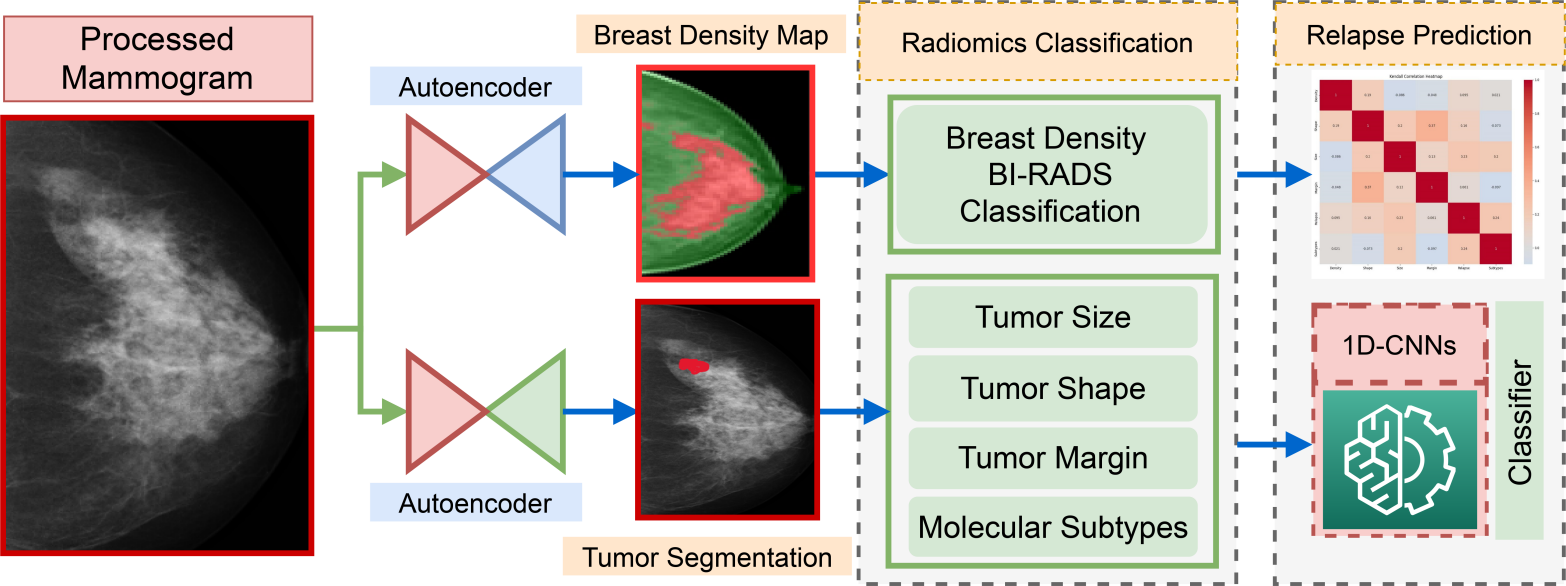
Overview of the proposed pipeline for radiomic feature extraction and integration in relapse prediction.

This work presents a comprehensive decision support system designed for BC relapse prediction by leveraging radiomic biomarkers extracted from digital mammograms as, shown in **Figure 1**. The framework incorporates tumor segmentation, BC subtypes classification, breast density mapping, and morphological analysis of the tumor regions to derive key features such as tumor shape, margin, size, and molecular subtype. These radiomic biomarkers, alongside BI-RADS-based breast density classification, are processed using a 1D-CNN model, demonstrating its ability to efficiently handle complex imaging data and enhance recurrence prediction accuracy.

## 2 Materials and methods

This study employed a comprehensive approach utilizing multimodal clinical data, which included mammographic images, radiologist-annotated tumor regions, and pathology-confirmed molecular subtypes. Tumor-related measurements—encompassing size, shape, and margins—were precisely extracted from standard diagnostic reports validated by experienced radiologists. While this study relies on expert-validated clinical annotations, recent developments in deep learning have demonstrated high accuracy in automated radiomic phenotype prediction, supporting future adoption of end-to-end computational pipelines.

### 2.1 Estimating breast density using deep learning models

Breast density estimation is critical for assessing BC risk and improving mammographic screening accuracy. Traditional approaches rely on radiologists’ visual assessments or semi-automated software, which can be subjective and inconsistent Park et al. (9). Deep Learning (DL), particularly Convolutional Neural Networks (CNNs), offers a more objective and standardized approach Saffari et al. (17). By training CNNs on labeled mammographic datasets (BI-RADS categories), these models can accurately identify patterns and textures associated with different density levels, reducing variability and outperforming conventional methods Khara et al. (14)Saffari et al. (17).

### 2.2 Extracting morphological features and molecular subtypes from tumor regions

Tumor morphology, including shape, size, and margins, plays a crucial role in characterizing breast cancer and assessing its aggressiveness Ma et al. (25) Gillies et al. (26). Traditional feature extraction methods rely on handcrafted techniques, which may overlook critical tumor characteristics SIN (2020). CNNs provide an automated and comprehensive approach by learning from annotated datasets to detect and segment tumor regions with high precision (27, 28). These models can uncover microtextural patterns linked to specific molecular subtypes, offering deeper insights into tumor behavior and treatment response Sutton et al. (29). Advanced techniques, such as multi-task learning and attention mechanisms, further enhance feature extraction by emphasizing the most relevant tumor regions. Recent advancements in radiomics and machine learning have enabled a more refined prediction of molecular subtypes, leveraging quantitative imaging biomarkers Yap et al. (30). CNNs efficiently capture spatial relationships and patterns, facilitating accurate classification of tumor morphology.

### 2.3 Breast cancer relapse prediction

**Figure 2:**
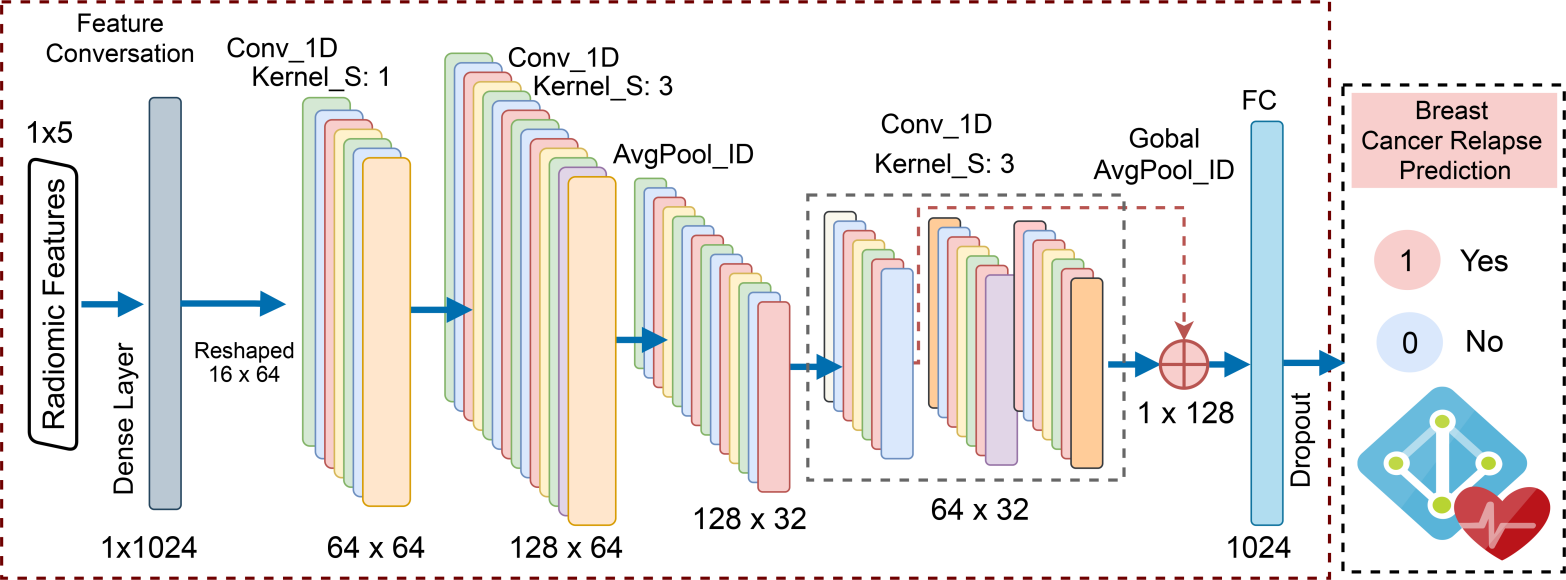
The proposed model architecture for relapse prediction.

In this work, we implemented a one-dimensional convolutional neural network (1-D CNN) tailored to the challenge of BC relapse prediction. As shown in the **Figure 2**, the model architecture initially emphasizes a streamlined process for converting features into high-dimensional representations to reflect better the spatial or sequential relationships within the underlying radiomics features Mathew et al. (31). The newly transformed features are then reshaped into a multi-channel pseudo-image format, effectively simulating a structured spatial domain. By expanding the feature representation to emulate structured spatial inputs, the network adapts to the morphological variability inherent in the dataset.

Further, deeper convolutional layers refine the learned features, providing robustness against variability in patient-specific tumor characteristics. Unlike traditional machine learning approaches such as Random Forest or XGBoost, which require extensive manual feature engineering, this deep learning framework learns these representations end-to-end, demonstrating adaptability to high-dimensional data. The final stages of the network incorporate a global pooling operation and fully connected layers with dropout for regularization, culminating in a sigmoid activation for binary relapse classification. This architecture integrates high-quality tumor features and leverages the inherent benefits of convolutional operations, such as spatial locality and feature abstraction.

#### 2.3.1 Training and evaluation

The proposed 1D-CNN model was trained on radiomic features, including Shape, Size, Margin, Density, and Tumor Molecular Subtype, automatically extracted from a private in-house dataset. Preprocessing steps included standardization, label encoding, and oversampling via Synthetic Minority Over-sampling Technique (SMOTE) to address class imbalance, ensuring robust predictive performance for the minority relapse class. The model was optimized using Binary Cross Entropy (BCE) Loss and the Adam optimizer with a learning rate of 0.001. The training was conducted over 200 epochs, with early stopping (patience = 25 epochs) applied based on validation loss to mitigate overfitting. Traditional machine learning models, including Random Forest, XGBoost, and MLP, were trained on the same features to benchmark performance. These models provided a comparative evaluation framework, validating the effectiveness of the proposed 1D-CNN in relapse prediction.

For evaluating the relapse model, we employed key metrics, Accuracy and Precision, to evaluate the positive predictions, Recall for its ability to identify all actual relapse cases effectively, and the F1-Score to balance precision and recall. The AUC-ROC measure True Positive Rate (TPR) against False Positive Rate (FPR) provides a comprehensive view of the model’s discrimination, critical for imbalanced classes. Additionally, we conducted SHAP (SHapley Additive exPlanations) analysis to determine the impact of individual radiomic features on the model’s predictions. This approach quantifies the importance of each feature, providing insight into which features have the most influence on the model’s output, leading to more informed refinements to the relapse model.

## 3 Results and discussion

### 3.1 Dataset

The dataset used in this study was developed in collaboration with our partner hospital, following ethical data collection and patient privacy guidelines. This dataset comprises clinical and pathological records for 148 BC patients, resulting in a total of 270 mammographic samples from both craniocaudal (CC) and mediolateral oblique (MLO) views, providing a robust foundation for our analysis. Our dataset includes detailed pathological annotations encompassing the following features. Molecular subtypes are classified into four categories: Luminal A (0), Luminal B (1), HER2-positive (2), and Triple-Negative (3). Tumor shape is categorized as oval (0), round or irregular (1), asymmetric (2), and distorted (3). Tumor margins are labeled as circumscribed (0), obscured or microlobulated (1), ill-defined (2), and spiculated (3). Breast density is recorded using the BI-RADS classification system, including entirely fatty (0), scattered fibroglandular tissue (1), heterogeneously dense (2), and extremely dense (3). Relapse status is encoded as a binary outcome, where 0 denotes no recurrence and 1 indicates documented relapse during the follow-up period. This comprehensive information is valuable for analyzing breast cancer characteristics and assessing relapse likelihood based on each patient’s unique tumor anatomy.

**Figure 3:**
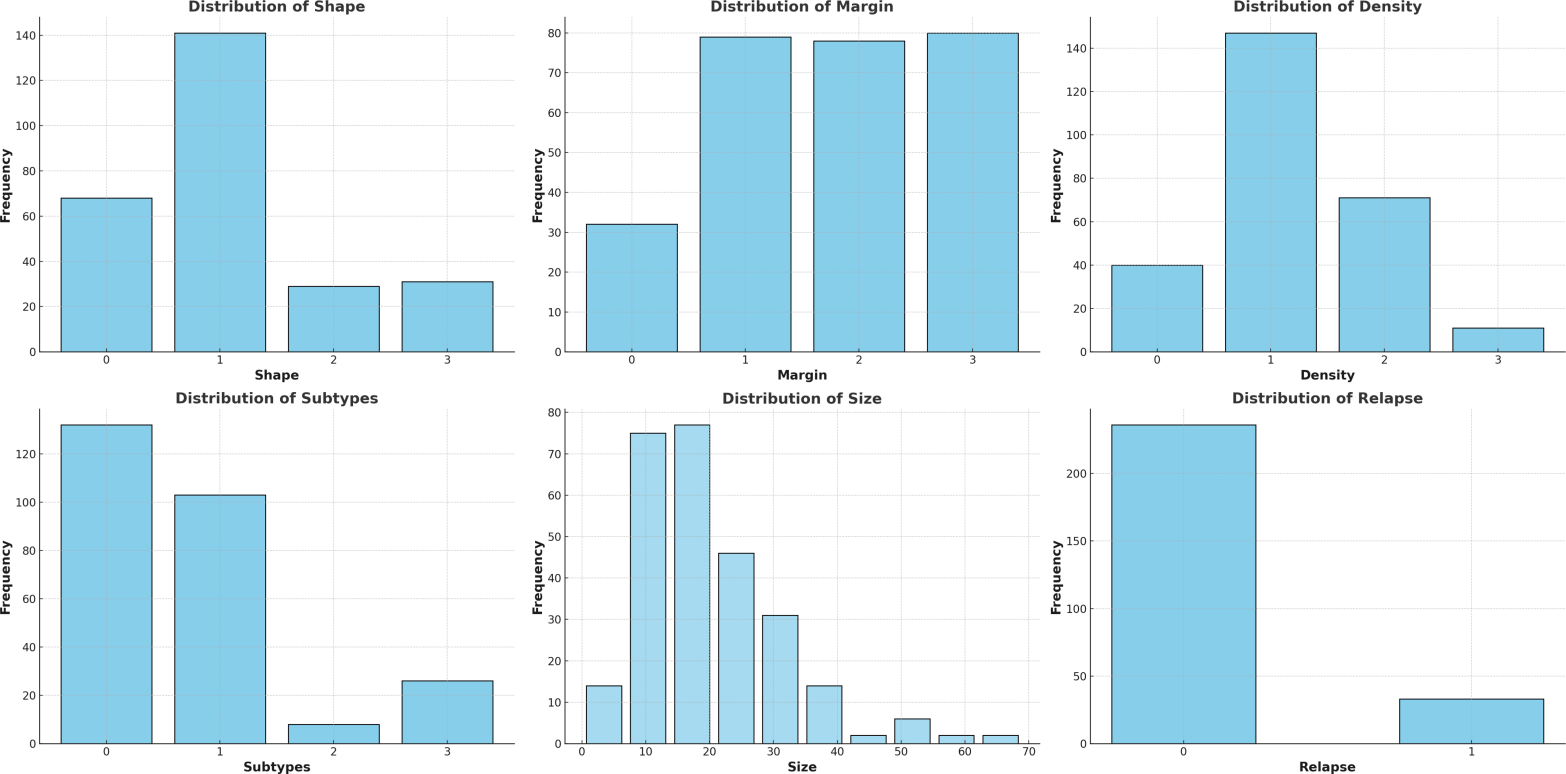
Descriptive statistics of the data using the histograms.

**Figure 3** provides a visual summary of the distribution of key breast cancer features in the dataset through six histograms. Breast density is predominantly classified as type 1 (scattered fibroglandular tissue), with smaller peaks at types 0 and 2, reflecting patterns common in screening populations. Tumor shape is most frequently type 1 (round or irregular), while types 0 (oval) and 3 (distorted) occur less often, and type 2 (asymmetric) is rare. Tumor margins are distributed primarily across non-circumscribed categories (types 1–3), with fewer circumscribed cases (type 0). Tumor sizes, measured in both CC and MLO views, follow a right-skewed distribution, with most lesions between 5 and 30 mm and a peak near 10 mm. Molecular subtypes are imbalanced, with Luminal A (0) and Luminal B (1) being the most common, while HER2-positive (2) and Triple-Negative (3) subtypes are less frequent but clinically more aggressive. Relapse cases are relatively few, introducing a moderate class imbalance that should be considered in downstream modeling.

**Figure 4:**
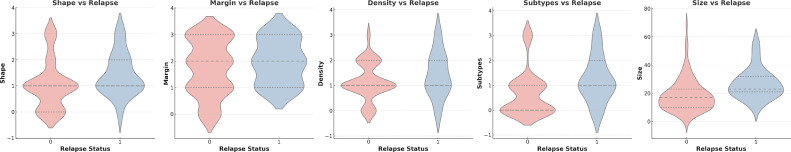
The plots show the distribution of tumor features by relapse status, highlighting associations with larger tumor size, aggressive subtypes, and irregular shapes and margins—features linked to relapse risk.

**Figure 5:**
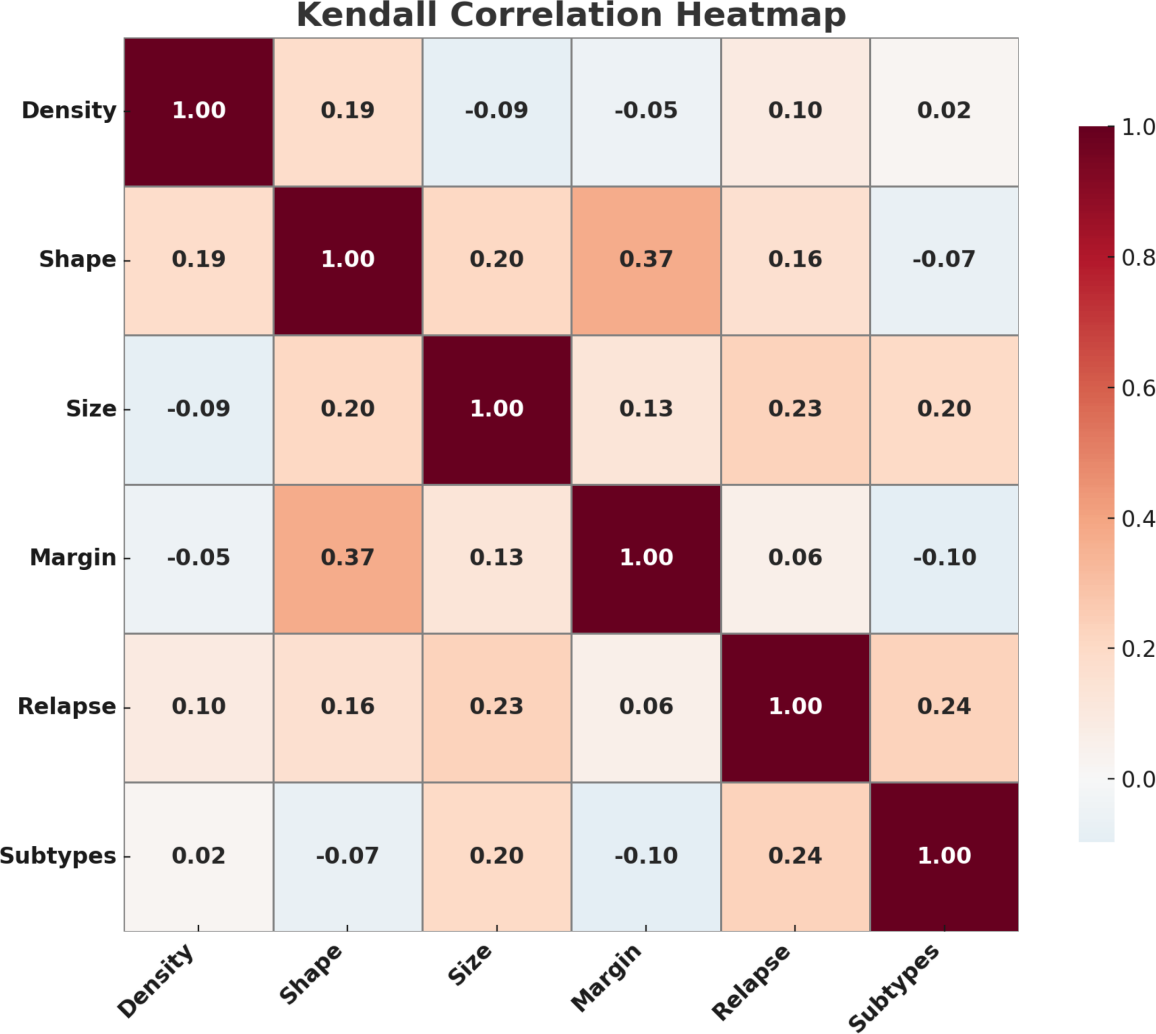
Correlation heatmap of breast cancer features, highlighting key associations such as shape–margin (*ρ* = 0.37) and subtypes–relapse (*ρ* = 0.24).

To explore the relationship between tumor characteristics and relapse, we examined feature distributions across relapse groups using violin plots (**Figure 4**). Patients who experienced relapse tended to have larger tumors, were more likely to present with aggressive molecular subtypes such as HER2-positive and Triple-Negative, and often exhibited irregular shapes and poorly defined margins—traits commonly associated with higher relapse risk. These patterns were further supported by Kendall’s rank correlation analysis (**Figure 5**), which showed moderate positive correlations between relapse and tumor size (*τ* = 0.23), as well as relapse and molecular subtype (*τ* = 0.24). A stronger correlation between tumor shape and margin (*τ* = 0.37) suggests a consistent association between morphological irregularity and invasive features. Some other feature pairs had near-zero correlations, suggesting minimal linear associations and pointing to the independence of many BC characteristics.

### 3.2 Classification performance

To predict BC relapse, we implemented a one-dimensional convolutional neural network (1D-CNN) model leveraging critical radiomic features extracted from digital mammograms. Additionally, a series of conventional machine learning models—including Random Forest, Support Vector Machines (SVM), Logistic Regression, and XGBoost—were systematically evaluated to establish comprehensive comparative insights and benchmark the performance of our CNN approach in **Table 1**.

**Table 1:** Results with 5-fold cross-validation. Values in parentheses indicate the 95% confidence intervals.

Model	Accuracy (%)	Precision	Recall	F1-Score	AUC
Random Forest	0.88 (0.84–0.92)	0.52 (0.42–0.63)	0.87 (0.75–0.99)	0.65 (0.55–0.74)	0.89 (0.90–0.97)
XGBoost	0.89 (0.85–0.93)	0.57 (0.38–0.77)	0.60 (0.44–0.75)	0.57 (0.43–0.72)	0.91 (0.87–0.95)
CatBoost	0.83 (0.78–0.88)	0.40 (0.29–0.51)	0.69 (0.50–0.87)	0.50 (0.38–0.62)	0.87 (0.77–0.96)
1D-CNN	**0.95 (0.89–0.98)**	**0.80 (0.55–0.96)**	**0.93 (0.83–1.00)**	**0.85 (0.70–0.93)**	**0.96 (0.94–0.98)**

Bold values indicate the best result in each column.

The results indicate that the 1D-CNN model achieves comparatively better predictive performance than the other models evaluated. Specifically, the CNN model achieved an average area under the receiver operating characteristic curve (AUC) of 0.9579, significantly surpassing the performance of Random Forest (AUC = 0.8986), XGBoost (AUC = 0.9116), CatBoost(AUC = 0.8672). Beyond AUC, critical evaluation metrics, including sensitivity, specificity, precision, recall, and F1-score (shown comprehensively in **Table 1**), consistently highlighted the superior robustness and reliability of the CNN approach compared to traditional machine learning methods.

We conducted both McNemar’s and DeLong’s statistical significance tests to evaluate pairwise differences in classification accuracy and AUC, respectively, to assess the performance advantage of our proposed 1D-CNN model rigorously. McNemar’s test revealed that the CNN model achieved statistically significant improvements in classification accuracy over all traditional machine learning models evaluated: Random Forest (*statistic* = 6.0, *p* = 0.0023), XGBoost (*statistic* = 8.0, *p <* 0.0001), and CatBoost (*statistic* = 4.0, *p <* 0.0001). Complementarily, DeLong’s test demonstrated that the CNN significantly outperformed CatBoost (*z* = -3.6436, *p* = 0.0008) and Random Forest (*z* = -1.5992, *p* = 0.0420) in terms of AUC. However, the difference between CNN and XGBoost was not statistically significant (*z* = -1.7748, *p* = 0.0608). Collectively, these results suggest a robust and statistically validated advantage of the CNN model in classification performance relative to conventional machine learning models.

The observed performance differences can be attributed primarily to the CNN architecture’s inherent capacity to capture complex spatial and sequential relationships within radiomic features. Although our model used only five fundamental radiomic features—tumor size, tumor shape, margin characteristics, molecular subtype, and breast density—the CNN architecture facilitated a highly effective conversion of these limited input features into sophisticated, high-dimensional representations. This capacity enabled CNN to better interpret subtle but critical patterns within the radiomic data, capturing interactions and non-linear relationships missed by conventional machine learning models.

**Figure 6:**
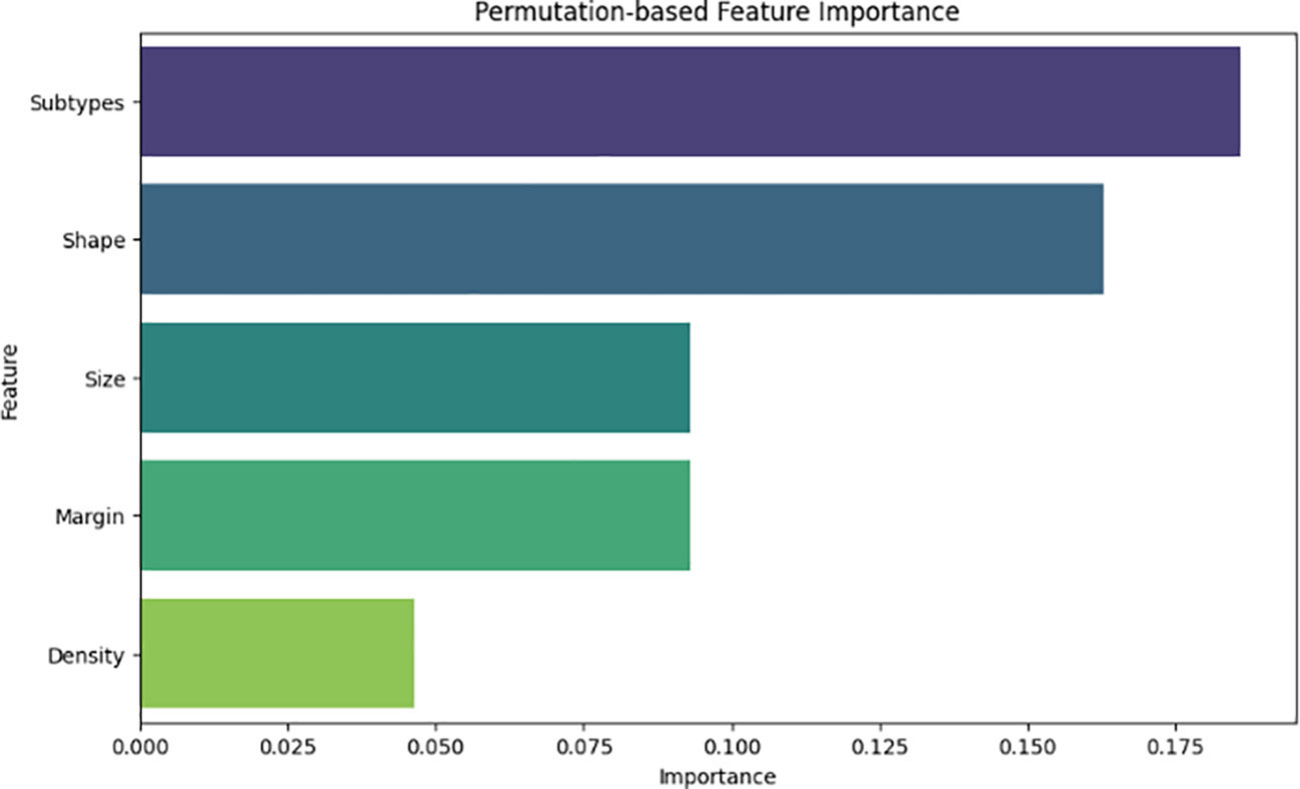
Feature importance analysis highlighting the most influential radiomic and clinical features contributing to BC relapse prediction.

We conducted comprehensive feature importance assessments and SHAP (SHapley Additive exPlanations) analysis to rigorously interpret and validate our predictive outcomes and further enhance clinical acceptability. As shown in **Figure 6**, the feature importance ranking highlighted molecular subtypes, tumor shape, and tumor size as the most critical predictive features associated with breast cancer relapse. The high significance of molecular subtypes aligns closely with existing clinical literature, as aggressive subtypes such as HER2-positive and Triple-Negative breast cancers (TNBC) are known to have a substantially higher tendency for recurrence due to their invasive biological behavior. Additionally, metastatic status at the time of initial diagnosis is a well-established, independent prognostic factor, particularly in HER2-positive BC patients, who exhibit significantly higher risk of relapse and worse survival outcomes due to the aggressive nature and high proliferative capacity of HER2-enriched tumors. Tumor shape also emerged as an essential predictive feature, supported by clinical evidence indicating that tumors exhibiting irregular shapes or spiculated margins typically correlate with greater malignancy potential, higher invasiveness, and consequently increased likelihood of future relapse.

**Figure 7:**
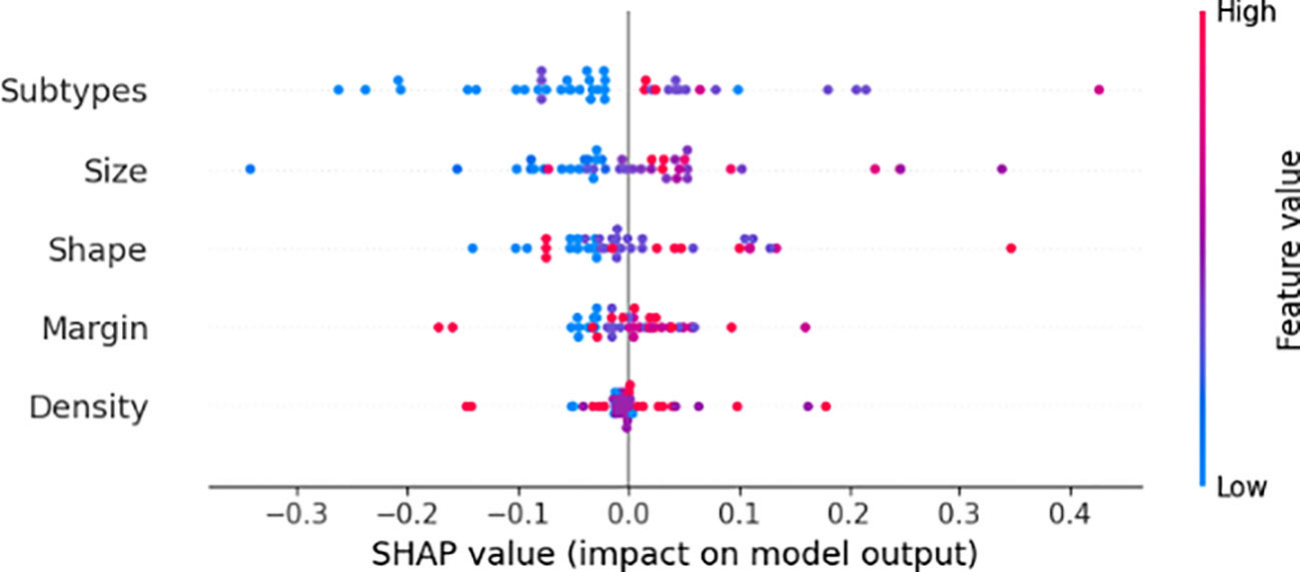
SHAP analysis illustrating the impact of these features on prediction.

**Figure 7** illustrates that SHAP analysis offered comprehensive insights into feature contributions, improving model interpretability. Notably, SHAP analysis demonstrated that molecular subtypes, particularly HER2-positive and Triple-Negative cases, had the strongest impact on relapse likelihood. Additionally, tumor shape irregularities and larger tumor size were positively associated with increased relapse risk, aligning with clinical evidence that these characteristics often indicate aggressive tumor behavior. These insights enrich clinical interpretability, providing a transparent understanding of essential predictive factors influencing relapse risk.

### 3.3 Limitation and future work

Our analysis focuses on confirmed mass cases, which represent approximately 80% of breast cancer cases. We excluded cases where the tumor was unclear or where high breast density obscured tumor visibility, as radiomic feature extraction in such instances demands additional imaging modalities like MRI or ultrasound for accurate tumor localization and classification. These modalities are crucial for identifying non-mass lesions and ensuring a more comprehensive relapse prediction framework.

To improve the generalizability of our relapse prediction framework, future work will incorporate data from multiple institutions that reflect variations in imaging protocols, patient demographics, and clinical settings. Integrating such heterogeneous data sources will facilitate broader applicability across real-world scenarios, including cases with high breast density and non-mass lesions. Moreover, the inclusion of multi-modal imaging, such as MRI and ultrasound, will support more accurate tumor characterization across a broader range of clinical presentations.

## 4 Conclusion

Integrating clinical and radiomic features plays a crucial role in breast cancer relapse prediction, as they capture key tumor characteristics linked to disease progression. Automated extraction of features like tumor shape, size, margin, and molecular subtypes enhances early relapse detection and predictive accuracy. Our proposed 1D-CNN model on these features outperformed conventional machine learning models, achieving an AUC of 0.95 by effectively learning complex feature relationships. SHAP analysis confirmed HER2-positive and Triple-Negative subtypes and tumor shape and size as key relapse predictors. This study highlights the potential of deep learning-driven radiomics for early, non-invasive relapse prediction, improving personalized treatment strategies. Future research should focus on validation in larger, multi-center datasets for broader clinical adoption.

## Data Availability

Data handling adhered to Protection of Personal Data standards, in compliance with Article 7.1 of the Organic Law 15/1999. Requests to access the datasets should be directed to hatem.abdellatif@urv.cat.
